# 

**Published:** 1874-01-01

**Authors:** 


### INDEX TO VOL. XV.

**A** PAGE

American Public Health Association ... 606
Anæsthetics ... 584
Anasarca in Children, F. K. Bailey, M. D ... 483
Albuminuria, Clinical Lecture on Chronic ... 16
Arsenic, Antidote to ... 25
Axioms, Dr. Joseph Bell's ... 26
Aubrey, John ... 26
Arthritis, Rheumatoid ... 49
Agassiz ... 92
American Med. Association, 317, 301, 249, 221, 120, 92
Alumni of the Chicago Medical College ... 149, 123
Anus, Imperforate, E. O. F. Roler, M. D ... 212
Association of American Medical Editors ... 305, 222
Archives of Electrology and Neurology ... 260
American Journal of Obstetrics ... 260
Amer. Ambul'e, History of, T. W. Evans, M. D ... 335
Astragalus, Dislocation of ... 461

**B**

Bubonocele, by F. K. Bailey, M. D ... 3
Bloodless Method of Operating in Mercy Hospital, Chicago, Trial of ... 12
Belladonna and Opium, Antagonistic effects of, by H. Wardner, M. D ... 79
Biliousness ... 98
Blood, Detection of, by the Spectrum Microscope ... 227
Berlin Notes, M. P. Hatfield ... 421, 278
Bowel Affections of Children ... 395
British Medical Association ... 469
Belladonna vs. Opium, O. T. Maxson, M. D ... 576

**C**

Cyclopedia of the Practice of Medicine, (Review) . 620
Cornea, Abscess of, treated by Kneading and Steam 601
Cerebral Disease, Obscure, Clinical Lecture, N. S. Davis, M. D ... 591
Croup, Clinical Lectures on, Dr. H. Roger ... 579
Croup in Relation to Tracheotomy, J. S. Cohen, M. D. (Review) ... 559
Cancer of the Breast, Excision by Scissor-Cutting . 554
Chloroform, the Condition of the Pupil during the administration of ... 542
Catarrh, Lecture, F. H. Davis ... 487
Chloroform, Apparent death from; recovery ... 479
Climate and Disease ... 416
Cutaneous Diseases, a rep. on, by H. Nance, M. D. 367
Correction ... 366
Clinical Notes, by F. K. Bailey, M. D ... 361
Chicago Journal of Nervous and Mental Disease, 259, 181, 13.
Chicago Society of Physicians and Surgeons, 578, 550, 529, 496, 466, 446, 423, 396, 323, 306, 271, 223, 171, 146, 119, 93, 70, 14.
Colorado as a Health Resort, by Chas. Denison ... 37
Cutaneous Subjects, Practical Notes on, by Tilbury Fox, M. D ... 476, 154, 76, 199
Cleft Palate ... 77
Cancer of the Skin, Development of ... 77
Clinic, Prof. Gunn's Surgical ... 93
Cerebral Disease, Clinic, by N. S. Davis, M. D ... 105
Chicago Medical College ... 222, 145, 122
Chloroform-Narcosis, Resuscitation from ... 128
Cholera, Blood and Dejections in ... 151
Cholagogues, Have we any, Geo. N. Fuller, M. D. 170, 157.
Cardiac and Renal Disease, Rare Form of, N. S. Davis, M. D ... 183
Cleft Palate, A New Operation for ... 229
Cæsarian Section ... 232
Chicago Medical Society, 492, 464, 428, 383, 350, 320, 277, 250.
Chicago Medico-Historical Society ... 415, 269
Calculi, Nine, taken from one Patient ... 289

**D**

Diseases of Infancy, Lectures on, by C. West, M. D. 311
Dry Cupping Instead of Taxis in Hernia ... 24
Diphtheria, Treatment of ... 25
Delirium Tremens, Clinic by N. S. Davis, M. D. 116, 59
Diphtheria Successfully Relieved by Laryngotomy, by H. A. Johnson, M. D ... 86
Diphtheria, Glandular Enlargement in ... 99
Dissection, Legalizing of ... 121
Diseases of Children, A Practical Treatise on, by J. F. Meigs ... 130
Diseases of Women, A Clinical History of, by Rob. Barnes, M. D ... 180
DeWitt County Medical Society ... 225
Diphtheria, Treatment of, by Cauterization ... 232
Dead, On the Cremation of the ... 376
Diseases of Women, A Practical Treatise on, by T. G. Thomas, (Review) ... 508

**E**

Examination of the Sick, A Lecture by N. S. Davis, M. D ... 561
Elastic Ligature, Cases Illustrating the Use of .. 481, 19
Electro-Therapeutics ... 22
Extraneous Matter in the Stomach, Accumulation of 23
Enuresis, Treatment of ... 25
Epilepsy, Chloride of Potassium in ... 25
Eye, Treatise on the Diseases of the, by J. S. Wells, 52
Electro-Therapeutics, Dr. Brunelli on ... 97
Empyema ... 168, 98
Electricity, Lectures on the Clinical Uses of ... 182
East Tennessee, Climate of ... 186

**F**

Foreign Bodies in the Stomach ... 15
Floating Kidneys, Cases of ... 21
Femur, Intentional Fracture of ... 23
Females, Report on Diseases of, by Hiram Nance, M. D ... 27
Furunculi, Abortive Treatment of ... 51
Fatty Degeneration, Clinic by N. S. Davis, M. D ... 59
Formulary, A Universal, by R. E. Griffith, M. D .. 156
Fistule, The History of a Royal ... 213
Fistula in Ano ... 329, 247
Food and Dietetics, by F. W. Panz, M. D ... 407

**G**

Galvanic Wire in Surgery ... 100
Gleanings from Camp and Hospital, by F. K. Bailey, M. D ... 261, 209, 108
Gelseminum, by C. D. Hodge, M. D ... 152
Galvano-Therapeutics ... 182
Gunshot Wounds of the Abdomen, The Nature of . 182
Goitre of Tuberculosis, The Symptomatic ... 220
Genito-Urinary Organs, A Treatise on Diseases of, by W. H. Van Buren, M. D. & E. L. Keys, M. D 333
Gleanings from the German, E. J. Doering, 597, 573, 546, 525, 490.
Gynæcological Clinic by T. D. Fitch ... 540

**H**

Hernia, Strangulated, C. Winne ... 411
Hard to Please ... 348
Heart, Valvular Disease of the, Lester Curtis ... 313
Heart, Fatty Degeneration of the ... 228
Hypertrophy, Pseudo-Muscular ... 228
Humerus, Dislocation of, by Edwin Powell, M. D ... 4
Hare-Lip, Operation for ... 24
Hæmorrhage, Post-Partem, On the Use of Electricity in, by Chas. W. Earle, M. D ... 53
Hoarseness, Chronic, Remedy for ... 101
Hydrocele and Hydro-Sarcocele, by A. Given, M. D 135
Hermann, Dr. A., Death of ... 145
Hæmorrhage, Restraint of During Operations in the Mouth, E. Andrews, M. D ... 169
Hand Book of Med. and Surg. Reference ... 182
Hæmorrhage, Post-Partem, Perchloride of Iron in . 203
Hair, in its Microscopical and Medico-Legal Aspect 226

**I**

Invermination, Associated with Measles, by F. K. Bailey, M. D ... 3
Ill's State Medical Society, Transact's of .. 248, 171, 78
Inguinal Hernia, Strangulated, Aspirating Puncture in ... 101
Intra-Uterine Injections, Danger of ... 144
Inflammation, New Researches on ... 152
Insomnia of Infants, Chloral Hydrate in ... 202
Insulation in the Treatment of Rheumatism and other Diseases ... 255
Intermittent Fever, Nervous Derangem't following . 290

PAGE

Ipecac, Study of the Therapeutical Use of..........436
Infant Diet, A. Jacobi, M. D. (Review)............508
Insanity, Puerperal, W. W. Godding, M. D........582

K

Koumys, Therapeutical Properties of..............461

L

Livingstone, Dr. His Fight with a Lion............527
Lipsomata, Means of Diagnosticating..............24
"Lancet" Jubilee....................................25
Lithotrity, Sir H. Thompson on....................100
Locomotor-Ataxy, Rest in..........................101
Lachrymal Apparatus, Diseases of, by L. L. Lambert, M. D..........................................131
Lupus, E. Andrews.................................247
Leucocyte and Pus Corpuscle.......................252
Ligation of Arteries, Manual by L. H. Farrabeuf, M. D.........................................311

M

Mushrooms, Poisoning by...........................457
Medical Congress, International...................414
Medical Colleges..............................326, 194
Medical Profession and Societies in Chicago.........44
Menstruation and Ovulation..........................50
Medical Jurisprudence, by A. S. Taylor..............78
Monster, A Double, like the Siamese Twins....433, 91
Midwifery, A System of, by W. Leishman, M. D..101
Midwifery, Manual of, by K. Schrœder, M. D.....104
Military Tract Medical Association................124
Med. Science, A Dict'y of, by R. Dunglison, M. D 130
Marine Hospital Service, Annual Report...........181
Military Tract Medical Association................382
Medical Societies and Representation..............500
Mortality in Paris, London, Lyons and Chicago Compared..........................................526
Menses, Occlusion with Retention of the, W. H. Byford............................................535
Medical Education. (Editorial)....................549

N

Nerves in Sebaceous Glands, The Termination of..151
Nightmare, A Cause of.............................153
Neuralgia, Intercostal in Women...................201
Nervous and Rheumatic Affections, Treatment of, by Static Electricity..............................205
Notes from Practice, by J. Schneck, M. D..........287

O

Ophthalmic Lecture, by S. J. Jones, M. D..........523
Ophthalmic Lectures by F. C. Hotz, M. D 595, 539, 521
Obstetrics, A Complete Handbook of (Review).....508
Obstetrical Forceps, A Modification of the, E. O. F. Roler..........................................459
Ophthalmic Miscellany........................409, 391
Ovulation and Menstruation....................267, 50
Obstetrics and Gynæcology, Report on Progress of..95
Obstetrical Journal of Great Britain and Ireland...208
Ovary, Multilocular Disease of, A. R. Jackson....235

P

Pleural Effusions, Operative Treatment of.........354
Pharmacy, A Treatise on...........................311
Pulmonary Cavities, On Local Treatment of...346, 265
Pleurisy, Artificial Rest in.......................256
Pus Corpuscles, Origin of, in Inflamed Cornea....254
Pathology of the Nervous System, The Study of, P. S. Hayes....................................240
Plagiarisms..................................413, 195
Pus, What it is Not, Lester Curtis, M. D..........165
Paracentesis Thoracis.............................155
Pneumatic Aspiration, Cases Illustrating the Use of.48
Private Medical Practice in New Orleans............72
Perineum, Case of Restoration of, by J. T. Everett, M. D.......................................83
Pneumonia, Treatment of (Translation) 218, 190, 137, 87
Parturition, Electricity in.........................99
Pemphigus..........................................99

PAGE

Perineum, On Lacerat's of, by Wm. Goodell, M. D 125
Psoriasis, Acetic Acid in..........................128
Perteet, A. J. Post-Mortem Appearance of the Body of...........................................134
Pericardial Sac, Aspiration of, by H. A. Johnson..517
Phthisis, On Strapping the Chest in...............530
Physiology of Man, by A. Flint, Jr. (Review)....534

Q

Quackery, Illustrations of the Literature of.......453

R

Rheumatism, Pericarditis, etc.....................587
Rhachitis and Mollities Ossium, Artificial Product. of 9
Rationalism in Medicine, by J. F. Todd, M. D......41
Renal Disease, by D. B. Trimble, M. D..............63
Regurgitation, A Case of Habitual, Simulating Rumination in the Lower Animals, by D. W. Graham, M. D........................................118
Rush Medical College..............................120

S

Surgical Emergencies, A Manual by W. P. Swain, (Review)........................................534
Secular Press and the Profession, T. D. Washburn, M. D......................................509
Sciatica...........................................485
Speculum, An Improved Form, by D. T. Nelson...419
Sex in Utero, On the Determining of..............385
Syphilis, Notes on. From *La France Medicale*.....6
State Medical Society, Transactions of.............13
Stricture of the Urethra, Dilation in..............24
Spinal Cord, Chronic Disease of.....................1
Strychnine, Poisoning by, Treated by Chloroform Inhalations.......................................51
Syphilis, Notes on, Translation....................66
Stone, Diagnosis of...............................100
Sphygmograph, The, by Edgar Holden, M. D......103
Sarcoma, Melanotic of the Choroid, F. C. Hotz, M. D.............................................112
Surgical Anatomy, The Student's Guide to, by E. Bellamy..........................................129
Syphilis and Dermatology, Notes on, by J. N. Hyde 140
Small-Pox, Statistics of, by H. Gradle, M. D......188
Social Evil, The...................................193
Stethoscope, Binaural, A New Form of, F. H. Davis............................................315
Syphilis, Its Analogy to a Disease of the Silk-Worm 337
Sugar in the Urine, An Improved Test for, W. S. Haines, M. D......................................569

T

To Subscribers.....................................13
Tape-Worm, by D. B. Trimble, M. D.................63
Theory and Practice of Medicine, by F. T. Roberts.78
Therapeutics, Materia Medica and Toxicology, A Treatise on, by H. C. Wood, Jr., M. D.........285
Toxicology, A Manual of, by J. J. Ruse. M. D....285
Transfusion, Direct, from Animal to Man, Two Cases............................................294
Tuberculosis, Consumption, etc. Lectures by L. Buhl, M. D. (Review)............................455
Typhoid Fever, Statistics of Mortality from.......550
Therapeutics and Materia Medica, by A. Stille, M. D. (Review)......................................559
Typhoid Fever, Anatomical Changes in, Klein.....618

U

United States, Climate of in Relation to Tuberculosis 47
Urticaria, Period'l, by J. Schneck, M. D...........65
Uterus, Spontaneous Expulsion of the..............155
Urethritis, Treatm't of, by Cubebs and Oleo-Resins 296
Urethra, Stricture of, The Electrical Treatment...502

V

Variola-Varicella Question..........................36

W

Women's Hospital Medical College. Commencem't 122
White Corpuscles, Migration of....................150
Wisconsin State Medical Society...................349

TO SUBSCRIBERS.—Our readers will notice some changes in the present number of THE EXAMINER. The size of the page has been lessened slightly, but retaining the double columns; and the number of pages have been increased to the extent of adding one-third more of reading matter. This adds several dollars to the cost of each number, and ought to induce every subscriber not only to pay his subscription promptly, but also to use his influence in inducing his neighboring practitioners to subscribe.

